# Dumbbell versus cable lateral raises for lateral deltoid hypertrophy: an experimental study

**DOI:** 10.3389/fphys.2025.1611468

**Published:** 2025-07-07

**Authors:** Stian Larsen, Milo Wolf, Brad J. Schoenfeld, Nordis Ø. Sandberg, Andrea B. Fredriksen, Benjamin S. Kristiansen, Roland van den Tillaar, Paul A. Swinton, Hallvard N. Falch

**Affiliations:** ^1^ Department of Sports Science and Physical Education, Nord University, Levanger, Norway; ^2^ Academy for Personal Training, Fredrikstad, Norway; ^3^ Applied Muscle Development Lab, Department of Exercise Science and Recreation, CUNY Lehman College, Bronx, NY, United States; ^4^ Department of Sport and Exercise, School of Health Sciences, Robert Gordon University, Aberdeen, United Kingdom

**Keywords:** resistance profile, muscle thickness, shoulder, within-participant design, Length-tension relationship

## Abstract

**Introduction:**

This study compared the effects of dumbbell versus cable lateral raises on lateral deltoid muscle thickness (MT) in resistance-trained men and women, with shoulder adduction/abduction range of motion standardised and matched between conditions.

**Methods:**

Twenty-four participants completed an eightweek intervention comprising two weekly resistance training sessions of five sets of lateral raises completed to momentary failure. The study employed a within-participant design with each participant’s arms randomly allocated to the cable or dumbbell lateral raise. MT of the proximal and distal lateral deltoid was assessed via B-mode ultrasound. Data were analysed in a Bayesian framework including both univariate and multivariate mixed effect models with random effects. Differences between conditions were estimated as average treatment effects, with inferences based on posterior distributions and Bayes Factors (BFs).

**Results:**

Results showed that lateral deltoid muscle thickness increased by 3.3%–4.6% during the intervention. Moreover, univariate analyses provided “moderate” support for the null hypothesis for both the distal (BF = 0.27) and proximal (BF = 0.22) lateral deltoid. Multivariate analysis provided “extreme” support for the null hypothesis (BF < 0.01). Within-intervention results indicated that conditions produced small or small to medium improvements based on resistance training specific thresholds.

**Conclusion:**

In conclusion, our data suggest that both dumbbell and cable lateral raises are similarly effective for increasing lateral deltoid muscle hypertrophy in resistance-trained lifters.

## Introduction

Resistance training is widely recognised as an effective strategy for promoting skeletal muscle hypertrophy in humans (Moreno et al., 2024). The influence of joint range of motion (ROM) on subsequent muscle growth has garnered significant attention in resistance training research (Pedrosa et al., 2022; Wolf et al., 2024; Kassiano et al., 2023b). Evidence suggests that training at longer-muscle lengths, whether through a lengthened partial ROM or a full ROM, may enhance hypertrophy in certain muscle groups, including the quadriceps femoris, biceps brachii, and triceps brachii (Kassiano et al., 2023a). However, this topic remains contentious (Moreno et al., 2024), as some studies report superior muscle growth when performing resistance training at longer-muscle lengths (Pedrosa et al., 2022; McMahon et al., 2014; Bloomquist et al., 2013; Sato et al., 2021; Maeo et al., 2020; Maeo et al., 2023; Larsen et al., 2024; Kubo et al., 2019), whereas others do not (Kubo et al., 2019; Pinto et al., 2012; Stasinaki et al., 2018). This discrepancy has been suggested to be due to hypertrophic benefits of loading muscles at relatively longer lengths, rather than only emphasising maximum muscle length in isolation.

It has been proposed that the effects of lengthened training on muscular adaptations may be muscle-specific (Ottinger et al., 2023). Specifically, muscles active on the descending limb of the length-tension curve are hypothesised to benefit from being trained at longer muscle lengths (Ottinger et al., 2023). In addition, the specific ROM employed in a resistance exercise could also influence factors such as muscle activation patterns (Visser et al., 1990; Mitsuya et al., 2023). Evidence suggests that regional hypertrophy (nonuniform growth across muscle regions) aligns with the same muscle regions that are most activated during a given exercise (Wakahara et al., 2013; Wakahara et al., 2017). These factors complicate efforts to explain why some studies have reported hypertrophic benefits from longer-muscle length training, whereas others do not (Kassiano et al., 2023a).

Although the influence of muscle length on muscular adaptations has been frequently studied over the past decade, most investigations compare different joint ROMs rather than varying resistance profiles (Nunes et al., 2020; Zabaleta-Korta et al., 2020). Resistance profile refers to how torque demands vary across a joint’s range of motion due to the interaction between external load and limb positioning (Ottinger et al., 2023). This affects the point at which peak loading occurs in the movement and may influence regional hypertrophy (Zabaleta-Korta et al., 2020). Nunes and colleagues (Nunes et al., 2020) examined the effects of resistance training using a cable with an ascending resistance profile versus a free weight preacher curl with a descending resistance profile on changes in biceps brachii muscle thickness in untrained men. After a 10-week intervention, their results showed no significant differences in biceps brachii muscle thickness between the two conditions, suggesting that the resistance profile did not influence biceps brachii hypertrophy. It should be noted that Nunes and colleagues (Nunes et al., 2020) assessed a single region of the biceps (50% humeral length), which may have overlooked potential regional differences in muscle growth. In contrast, Zabaleta-Korta et al. (2023) found that different resistance profiles elicited region-specific hypertrophy in trained women following 9-week of elbow flexor training with preacher curls, which place peak torque at more extended elbow angles. Results indicated growth of only the distal region of the biceps brachii. As such, it was suggested that different regions of a muscle may grow in response to exercises that impose the greatest stimulus at specific points in the range of motion. However, as noted, caution is warranted when generalising findings from one muscle group to another (Ottinger et al., 2023). Therefore, further research into the effects of resistance profiles on other muscle groups is warranted.

Training programs frequently target the lateral deltoid, yet exercise prescription for this muscle often relies on surface electromyography (sEMG) studies (Coratella et al., 2020) or findings extrapolated from research on other muscle groups. For example, evidence indicates that both the cable and dumbbell lateral raise elicit greater sEMG amplitude than the shoulder press, with no differences between the conditions (Botton et al., 2013). However, the dumbbell lateral raise features an ascending concentric resistance profile with reduced torque when the lateral deltoid is in a lengthened position. In contrast, the cable lateral raise can be modified to provide a larger torque when the lateral deltoid is lengthened, creating a descending concentric resistance profile. Notably, the lateral deltoid is estimated to reach the descending limb of the length-tension relationship when the humerus is parallel to the torso (Garner and Pandy, 2003). Based on this observation, it is speculated that the lateral deltoid may benefit from being trained with a descending resistance profile, as this may enhance tension on the muscle in a more lengthened position, as opposed to maximising torque at shorter muscle lengths, such as in the dumbbell lateral raise. Therefore, this study aimed to compare the effects of a dumbbell versus a cable lateral raise on lateral deltoid muscle thickness in resistance-trained men and women, with shoulder adduction/abduction ROM standardised and matched between conditions. It was hypothesised that the cable lateral raise would result in more favourable hypertrophic outcomes, as its descending resistance profile provides peak torque around the muscle’s longest length, whereas the dumbbell lateral raise, with its ascending resistance profile, provides peak torque around the muscle’s shortest length.

## Methods

### Participants

**FIGURE 1:**
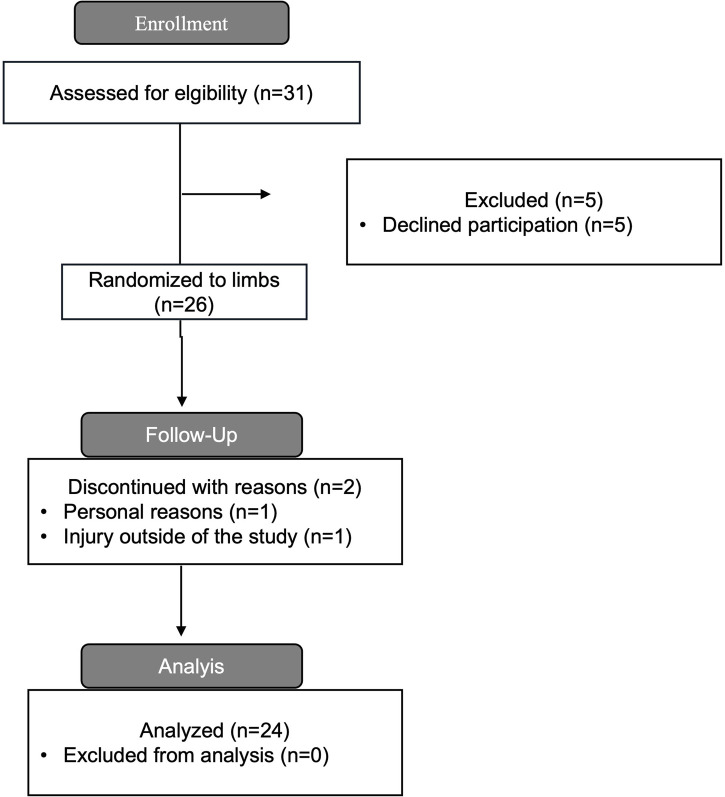
CONSORT diagram that shows the data collection process.

The sample size was determined based on prior Bayesian sample size calculations conducted by our group (Larsen et al., 2024), considering the within-participant design and multiple pre- and post-intervention measurements. This Bayesian framework prioritised describing probabilities and plausible values over dichotomous null hypothesis testing (Kruschke and Liddell, 2018). The within-participant design with informed priors and use of multiple pre- and post-intervention measurements enabled greater precision of estimates by controlling for individual factors such as genetics and lifestyle (MacInnis et al., 2017). Given that our research question addressed hypertrophy rather than strength, the cross-education effect which is reported to occur with unilateral training was not considered a confounding variable (Manca et al., 2017). To determine sample size we used simulation-based calibration of Bayes factors and assessed our ability to provide support for the correct hypothesis with sample sizes of n = 30 and n = 25 to align with our likely resource constraints. Priors were derived from meta-analyses and similar studies from our group (Larsen et al., 2024; Swinton et al., 2022; Wolf et al., 2023). The priors set on a standardized scale, included distributions for typical improvement N(0.44,0.40^2^), average treatment effect N(0.30,0.27^2^), heterogeneous response N(0,0.15^2^), and measurement error N(0,0.20^2^). Simulation-based calibration of Bayes factors was fit across 500 iterations using an average treatment effect of zero (no intervention difference), or from our non-zero distribution, each 50% of the time. The average posterior model probability for n = 30 and n = 25 was 49.7 (95%CrI: 41.2%–55.9%) and 48.6 (95%CrI: 40.0%–56.2%). The average percentage of posterior allocated to the alternative hypothesis when it was true was 84% and 81%, respectively for the two sample sizes. We judged these results to provide appropriate assessment of strength of evidence and attempted to recruit 30 participants, ultimately resulting in 26 which were included and 24 that completed the intervention (see Table 1; Figure 1). This number of participants is also consistent with many resistance training interventions comparing resistance training, and greater than many comparative within-participant designs in the field (Larsen et al., 2024; Refalo et al., 2024).

**TABLE 1:** Descriptive summaries of participant characteristics and current training load.

	Men (n = 16)		Women (n = 8)	
Variables	Mean (SD)	Range	Mean (SD)	Range
Age (years)	29.3 ± 5.8	22–41	25.3 ± 3.2	21–32
Body mass (kg)	85.1 ± 14.1	-	71.6 ± 15.1	-
Height (cm)	178.6 ± 7.2	168–197	164.9 ± 6.6	160–174
Lateral raise set volume (weekly)	5.5 ± 3.6	0–12	4.6 ± 1.7	3–7.5
Shoulder press set volume (weekly)	3.8 ± 2.2	0–6	3.6 ± 2.6	0–7.5
Lateral deltoid set volume (weekly)^a^	7.4 ± 4.0	2–15	6.4 ± 2.5	3–11
Resistance training experience (years)	7.4 ± 3.9	3–16	6.8 ± 2.5	4–11
Resistance training frequency (weekly)	4 ± 0.8	3–5.5	3 ± 0.9	2–4.5
Lateral deltoid frequency (weekly)	1.8 ± 0.5	1–2.5	1.7 ± 0.6	1–2.5

^a^ Weekly lateral deltoid training volume is calculated as the sum of sets from lateral raises and shoulder presses, with each lateral raise set counted as 1 set and each shoulder press set contributing 0.5 set toward the lateral deltoid.

Participants were instructed to maintain their habitual dietary patterns throughout the intervention, with a recommendation to modestly increase caloric intake by consuming larger portions than usual. Also, participants were encouraged to achieve a daily protein intake of at least 1.6 g per kilogram of body mass (Morton et al., 2018). Body mass was measured pre- and post-intervention using a Tanita scale (MC-780MA, Riga, Latvia). Study procedures were performed in accordance with the latest revision of the Helsinki declaration. Ethical approval for the project was obtained from Norwegian Agency for Shared Services in Education and Research (ref number: 578,814). Furthermore, the study was submitted to the Regional Ethical Committee, which deemed the research project exempt from full representation (ref number: 795,724).

The study inclusion criteria were: 1) age range between 18 and 50 years old; 2) no self-reported previous or present use of anabolic steroids or illegal performance-enhancing agents; 3) consistently participated at least twice a week in resistance training for at least 3 years prior to the study; and 4) no injuries or illness that could hinder resistance training program performance or adherence (see Figure 1).

### Risk of confounding variables and bias

The study aim, hypothesis, and methods were preregistered on the Open Science Framework (osf.io/zmkhw) prior to data collection. To minimize potential biases, the study adhered to the Standards Method for Assessment of Resistance Training in Longitudinal Design (SMART-LD) checklist (see Supplementary file 1) (Schoenfeld et al., 2023). Additionally, we standardized sets, repetition number, shoulder abduction ROM, proximity-to-failure, rest intervals, and lifting durations in both the eccentric and concentric phase as each variable may influence the resistance training stimulus and potentially confound a causal relationship (Coratella, 2022).

### Resistance training procedures

Participants’ upper limbs were randomly assigned to one of two exercise conditions: (1) dumbbell lateral raises, or (2) cable lateral raises, using www.randomizer.org. Additional exercises (leg presses and calf raises) were standardised but performed separately. Randomisation was concealed from investigators and participants prior to the intervention.

**FIGURE 2:**
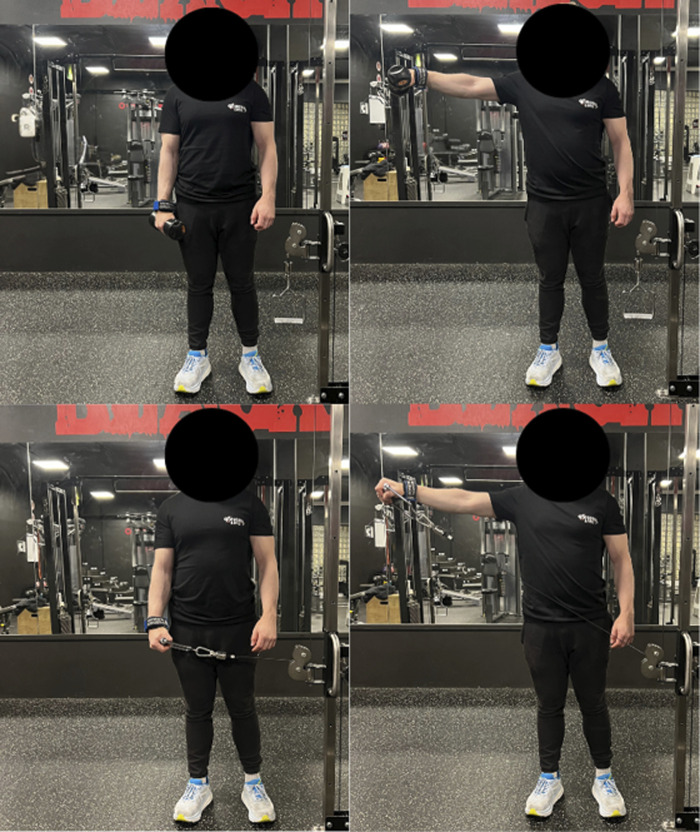
Illustration of the two lateral raises conditions trained with.

Participants performed four unilateral sets of lateral raises for each limb in the first week, totalling eight weekly sets. For the remaining 7 weeks, volume was increased to five unilateral sets of lateral raises for each limb per session, performed twice weekly, totalling ten weekly sets. Four participants self-reported that they trained with more than 10 weekly sets targeting the lateral deltoid muscle, whereas the remaining 20 participants increased their weekly lateral deltoid set volume during the intervention. Training sessions were performed on non-consecutive days for 8 weeks, for a total of sixteen training sessions. A familiarisation session established participants’ 16-repetition maximum (RM). Throughout the intervention, sets were performed to momentary failure (defined as inability to achieve 90° shoulder abduction, see Figure 2) with a target repetition range of 12–16 RM. Load adjustments followed a double progression strategy: if participants exceeded 16 repetitions in a set, the load was increased by 0.25 kg, while a decrease of 0.25 kg was applied if repetitions fell below 12. Participants were permitted to perform a self-selected warm-up. Rest intervals were ∼30 s between limbs and >90 s between sets for the same limb, with the order of limb training alternating weekly.

Participants were provided with an optional resistance training program that included Romanian deadlifts, exercises for the upper arms, horizontally loaded pectoralis major exercises, lat pulldowns, and seated rows with a narrow grip (see Supplementary file 1). This optional programme was included to accommodate resistance-trained participants and ensure adherence to the primary resistance training protocol in an ecologically valid framework. However, participants were instructed to avoid exercises that could confound lateral deltoid hypertrophy outcomes, including shoulder press variations, front raises, lateral raise variations, or incline bench press/fly variations above a 30° incline, as vertically loaded exercises have been shown to enhance lateral deltoid sEMG amplitude (Botton et al., 2013).

The eccentric and concentric phases of each lift were standardized to approximately 1 s each to try to match shoulder abduction and adduction angular acceleration, which could impact the torque. Participants were instructed to pause briefly at full shoulder adduction but not at full shoulder abduction. Shoulder abduction ROM was standardized and kept similar between conditions. Specifically, in both conditions, shoulder abduction and adduction were performed in the frontal plane, with an abduction ROM from the humerus being parallel to the body to an abduction angle of 90° (see Figure 2). Participants were instructed to perform abductions strictly in the frontal plane with a fully extended elbow joint. Some participants reported pain in the glenohumeral and/or scapulothoracic joints during strict shoulder abduction lateral raises. In these cases, they were permitted to perform shoulder elevation mid-way between the frontal and scapular plane. For these participants, the rotation angle of the humerus was kept consistent across both lateral raise conditions. The technical requirements were practiced during the familiarisation session using a goniometer and stopwatch, and subsequently monitored visually by a researcher throughout the intervention to ensure consistent execution and concentric–eccentric phase durations. Cable lateral raises were performed unilaterally with a Gymleco 225 cable crossover multi gym unit (Gymleco, Eskilstuna, Sweden), whereas dumbbell lateral raises were performed unilaterally with Impulse dumbbells (Impulse Fitness, Jimo, Qingdao, China). The cable height was adjusted so that the pulley stack was positioned approximately horizontally aligned with the hand, imposing peak adduction torque at peak shoulder adduction ROM. However, due to a 24 cm distance between the machine’s attachment points, a perfectly horizontal alignment was not always achievable. All participants performed lateral raises for both conditions with Versa Gripps - Pro series (Versa Gripps, Hancock, Maine, United States) to ensure that limited grip strength did not negatively affect hypertrophic outcomes for both lateral raise conditions.

All sessions were supervised by at least one trainer, all of whom held at minimum a bachelor’s degree in sports science. Supervisors received detailed instructions regarding the resistance training procedures, and two pilot tests were conducted to ensure they were familiar with the procedures. Both the data collection and intervention were conducted at a local training center.

### Muscle thickness assessments

Muscle thickness was assessed using b-mode ultrasonography (Echo Wave 2 Software; Telemed, Latvia) with a 60-mm probe operating at 9 MHz with Chemolan transmission gel (Chemodis, DA, Alkmaar). Participants were instructed to refrain from resistance training or other strenuous physical activity for 72 h prior to the measurements. We employed ultrasound for this study because of its high concurrent-validity compared to the gold standard, magnetic resonance imaging (Reeves et al., 2004).

**FIGURE 3:**
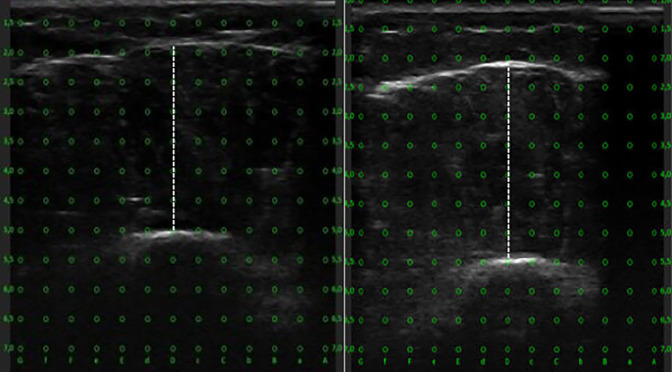
Representative ultrasound images of the lateral deltoid muscle measuring muscle thickness. The left panel shows the proximal region, and the right panel shows the distal region.

To our knowledge, no prior resistance training study has directly assessed muscle thickness in the lateral deltoid muscle. To assess reliability, a test was conducted with five participants. Measurements of the lateral deltoid were taken at 25%, 35%, 40% and 45% of the length with a straight line between the acromion to the lateral epicondyle of the humerus, following a procedure similar to that of Bhansing, Van Rosmalen (Bhansing et al., 2015) that measured fascial thickness of the lateral deltoid at 25% between the acromion and lateral epicondyle. Pilot testing yielded typical errors below 0.4 mm and coefficient of variations (CV) below 2.2% and aided in selecting measurements at 25% and 40% as these provided the most reliable measurements (See Figure 3).

Ultrasound measurements were conducted during two distinct baseline tests and two post-intervention tests, with at least 24 h separating the repeated tests. Upon arrival at the laboratory, participants adopted a supinated position on a bench and rested for 10 min before the ultrasonography began. Transverse images of the lateral deltoid were captured between the edge of the humerus and the superficial aponeurosis of the lateral deltoid. During the ultrasound procedures, the pressure from the linear transducer against the skin was minimized. Additionally, three images were taken from each site of the lateral deltoid and averaged at both baseline tests and postintervention tests. If a difference greater than 10% was observed, a fourth image was taken. Reliability measures between the two baseline tests and the two post intervention tests showed typical error <0.47 mm, intraclass correlations (ICC) > 0.98, and CV of <2.3% for the distal lateral deltoid and typical error <0.45 mm, ICC >0.98, and CV <2.3% for the proximal lateral deltoid. To ensure precise replication of the measurement sites at post-intervention testing, the anatomical landmarks and skin markings used during the pretest were documented by photographing the marked lines. These images were stored on a password-protected memory device accessible only to the sonographers. Muscle thickness measurements were performed manually using ultrasound imaging software. Full blinding of both sonographers was not possible, as one of them was responsible for supervising several of the training sessions.

### Statistics

All analyses were conducted in R (version 4.4.0) within a Bayesian framework. Analyses were conducted using a multivariate and separate univariate linear mixed effects models model, with random effects allocated to account for repeated measures and the within participant design (Magezi, 2015). The primary estimate for the study was the difference in hypertrophy induced by training with cable versus dumbbell lateral raises. The estimator used was the average treatment effect (ATE), defined as the mean difference in muscle thickness change scores between the limbs.

Within-condition treatment effects were also quantified to evaluate the overall effectiveness of each intervention independently and compared to thresholds specific to strength and conditioning (Swinton et al., 2022). Inferences were based on: 1) the posterior distribution of the ATE estimates and their corresponding credible intervals; and 2) Bayes factors to quantify the strength of evidence for either a non-zero ATE (alternative hypothesis H_1_) or a zero ATE (null hypothesis H_0_). Standard qualitative labels for interpreting the strength of evidence were applied (Lee and Wagenmakers, 2014). The analyses were performed using the *brms* R package interfaced with Stan to perform sampling (Bürkner, 2017). Bayes factors were estimated using the bridge sampling algorithm (Gronau et al., 2020).

A comprehensive Bayesian workflow was adopted for the analysis and comprised: 1) use of informative priors derived from meta-analyses in the field (Swinton et al., 2022); 2) evaluation of prior appropriateness through prior predictive checks; 3) running models and assessing the stability of estimates via repeated iterations with the same data; 4) evaluation of posterior distributions through posterior predictive checks and sensitivity analyses with non-informative priors; and 5) simulation-based calibration of Bayes factors (Schad et al., 2023). To enhance accuracy, transparency and replicability, the WAMBS-checklist (When to worry and how to Avoid Misuse of Bayesian Statistics) was followed (Depaoli and Van de Schoot, 2017). Summaries of the Bayesian workflow, including prior and posterior evaluations, are reported in Supplementary file 3.

## Results

### Attendance

Twenty-four of the 26 participants completed the intervention and were included in the analyses. One participant withdrew due to an injury unrelated to the study while another withdrew for personal reasons. Mean attendance was 15.4 out of 16 resistance training sessions, representing a compliance rate of over 95%. Body mass increased for 23 out of 24 participants (males: 2.3 ± 1.2 kg; range 0.9–5.8 kg; females 2.1 ± 1.4 kg; range −0.2–7.8 kg), indicating adherence to an energy surplus across the interventional period.

### Hypertrophy

**FIGURE 4:**
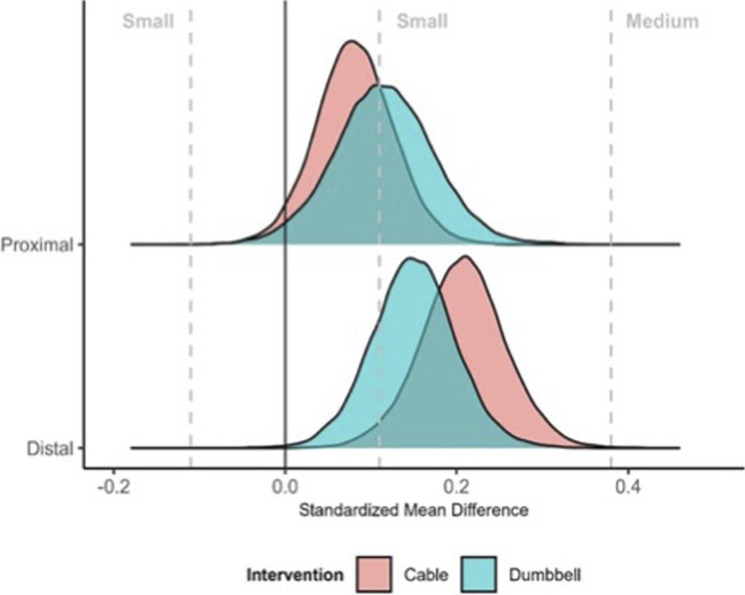
Standardized mean difference estimates of change in muscle thickness during the intervention. Densities illustrate posterior density estimates of within-intervention changes standardized relative to group baseline standard deviation.

Descriptive summaries of the sample data are presented in Table 2. Univariate analyses of the ATE identified “moderate” evidence in support of the null hypothesis for both the distal (Bayes factor = 0.27) and proximal (Bayes factor = 0.22) lateral deltoid. Posterior estimates of the ATE_Cable:Dumbbell_ were −0.25 (95%CrI: 0.72 to 0.22 mm) for the distal lateral deltoid and 0.20 (95%CrI: 0.57–0.94 mm) for the proximal lateral deltoid. Combining the regions within a multivariate analysis resulted in similar ATE estimates and provided “extreme” evidence in support of the null hypothesis (Bayes factor<0.01). Within-condition analyses using standardized mean difference estimates indicated that interventions were likely to produce small or small to medium improvements (see Figure 4). Output from the WAMBS checklist and Bayes factor simulation-based calibration are presented in the supplementary file and identified no concerns with the analyses.

**TABLE 2:** Descriptive summary of pre- and post-intervention values (mean ± SD).

	Cable (n = 24)			Dumbbell (n = 24)		
Variable	Pre	Post	Δ%	Pre	Post	Δ%
Lateral deltoid distal (mm)	21.5 ± 4.3	22.5 ± 4.4	4.6	21.9 ± 3.9	22.7 ± 4.0	3.9
Lateral deltoid 40 proximal (mm)	18.6 ± 5.8	19.1 ± 5.7	3.3	18.8 ± 6.0	19.4 ± 6.4	3.4

### Repetition volume

**FIGURE 5:**
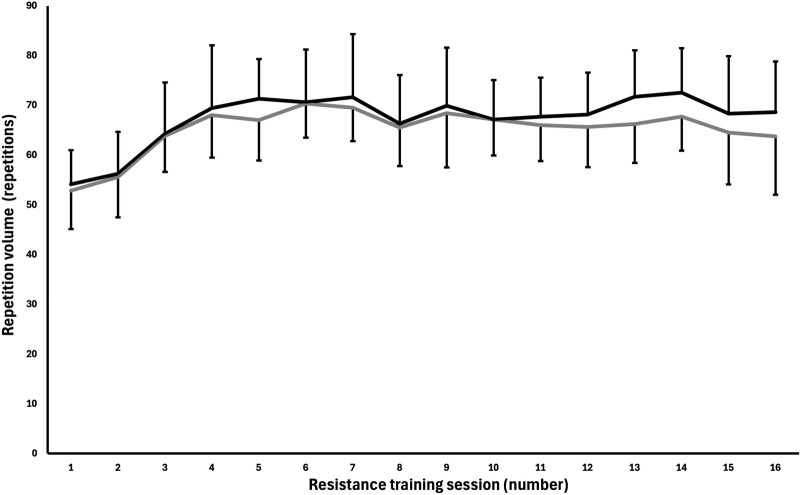
Mean ± SD repetition volume lifted each resistance training session for the cable and dumbbell group in the unilateral lateral raise exercise. Black = dumbbell group. Grey = cable group.

The mean repetition volume lifted during the intervention was 65.1 ± 8.2 repetitions for the cable group and 67.4 ± 9.7 repetitions for the dumbbell group per session. The repetition volume in sessions 1 and 2 was 52.8 ± 7.7 and 55.5 ± 8.0 repetitions for the cable group, and 54.1 ± 7.9 and 56.2 ± 8.5 repetitions for the dumbbell group. When the number of sets increased in week two, the repetition volume was 63.8 ± 7.2 and 62.4 ± 10.4 repetitions for the cable and dumbbell groups. In the last RT session, repetition volume was 63.7 ± 11.7 and 68.6 ± 10.2 repetitions for the cable and dumbbell groups (see Figure 5).

## Discussion

The primary aim of this study was to compare the effects of dumbbell and cable lateral raises on lateral deltoid muscle hypertrophy among resistance-trained men and women. Contrary to our research hypothesis, moderate evidence in favour of the null hypothesis was obtained for both the distal and proximal regions of the lateral deltoid. Within-condition treatment effects suggested that both conditions elicited small to medium increases in lateral deltoid muscle thickness with percentage changes ranging from 3.3% to 4.6% across assessed sites. Thus, our findings suggest that both dumbbells and cables present similarly effective options for eliciting hypertrophy of the lateral deltoid in resistance-trained men and women.

To our knowledge, this is the first study to investigate the effect of resistance profile on muscle thickness in the lateral deltoid; therefore, direct comparisons with previous studies are limited. However, our findings align with the hypertrophic outcomes reported by Wolf and colleagues (Wolf et al., 2024) (range: 4.5%–8.7%), who reported no advantage of lengthened partials compared to full ROM exercises for muscle thickness changes in the elbow flexors and extensors in resistance-trained participants following an 8-week training intervention. While the study by Wolf and colleagues (Wolf et al.) manipulated ROM and ours manipulated resistance profile, both approaches involve emphasising loading at relatively longer muscle lengths. This consistency suggests that trained individuals may exhibit limited responsiveness to variations in ROMs.

No region-specific differences were observed between the dumbbell and cable conditions, indicating a similar increase in lateral deltoid muscle thickness for both conditions. Non-uniform changes in muscle size is a phenomenon termed region-specific hypertrophy, which has been consistently demonstrated in resistance training research (Nunes et al., 2024). Proposed mechanisms for region specific hypertrophy include non-uniform muscle activation (Wakahara et al., 2013), variations in muscle pennation angle, and localized strain across muscle regions (Zabaleta-Korta et al., 2020). Region specific hypertrophy has been reported in other muscle groups, such as the biceps femoris short head when increasing torque when the muscle is in a lengthened position (Maeo et al., 2024). However, contrasting findings by Nunes et al. (2020) showed no significant differences in hypertrophy for the biceps brachii regardless of the range of motion in which torque was emphasized. These results highlight the complexity and variability of hypertrophic adaptations across different muscles and training modalities.

Training at longer muscle lengths has been proposed to enhance hypertrophy (Wolf et al., 2023), and therefore we speculated that the cable lateral raise descending resistance profile would augment muscle growth compared to dumbbells. However, the comparable improvements observed between conditions in this study could potentially reflect an insufficient emphasis on the stretched position. The standardized ROM in both conditions likely limited the activation of mechanosensing pathways, such as those speculated to be mediated by titin (Ottinger et al., 2023; Wackerhage et al., 2019). Compared to dumbbells, a potential advantage of performing the cable lateral raise is the ability to increase shoulder adduction ROM while maintaining tension throughout the movement. Since our study standardized shoulder ROM and manipulated only the resistance profile, future research should compare the lateral raise exercise performed with dumbbells to those with cables, allowing for an increased shoulder adduction ROM in the cable condition.

Although participants were instructed to maintain a similar lifting tempo during repetition performance, we observed that towards the end of the sets, they increased acceleration out of the bottom position for both conditions. While the external moment arms differ between dumbbells and cables, the resistance profile in terms of shoulder adduction torque are influenced by the force production component, as force equals mass multiplied by acceleration. Consequently, shoulder torque may still be present with dumbbells during lower shoulder abduction angles, even though the moment arm is shorter compared to cables. Thus, it is speculated that the lateral deltoid may experience sufficient loading at adequate muscle lengths during dumbbell lateral raises, potentially helping to explain why comparable muscle hypertrophy of the lateral deltoid was observed between the two conditions.

### Limitations

This study has several limitations that warrant consideration. First, the advanced training status of the participants likely contributed to the modest hypertrophic adaptations observed, as resistance-trained individuals experience slower rates of muscle growth compared to untrained populations. This limitation was compounded by the relatively short intervention duration (8 weeks), which may have constrained the ability to detect meaningful differences between conditions. In addition, the importance of training intensity of effort for increasing hypertrophic stimulus on a set-per-set basis (Robinson et al., 2024) and influence of training volume (Pelland et al., 2024), which were matched between the two conditions, are speculated to be greater contributors to muscle hypertrophy. Second, the typical error (around 0.4 mm) associated with ultrasound measurements was relatively large compared to the observed changes in muscle thickness (0.5–1 mm), potentially reducing the power to detect small average treatment effects. Third, while body mass was regularly monitored, and most participants were in an energy surplus during the intervention, nutrition was not systematically tracked, which may have introduced variability in training responses. Fourth, the participants were allowed to train other exercises but no exercises that targeted the lateral deltoid as the prime mover. Fifth, although we utilized a within-subject design, no control group was included which limits the ability to quantify the degree of measurement error accurately over time (Hammert et al., 2024). Finally, ultrasound measurements were taken at only two sites on the lateral deltoid, whereas regional differences in muscle thickness increases may have occurred at other sites.

### Practical applications

The results of this study demonstrate that both dumbbell and cable lateral raises can promote lateral deltoid hypertrophy in resistance-trained men and women. The comparable hypertrophy outcomes observed suggest that both variations can be employed for training the lateral deltoid based on individual preference.

## Conclusion

This study provides evidence of comparable hypertrophy in the lateral deltoid following 8 weeks of training with either dumbbell or cable lateral raises in resistance-trained individuals. However, the short duration and the advanced training status of the participants may have limited the ability to identify any systematic differences in hypertrophic adaptations between the conditions. Thus, future studies should try to replicate these findings with longer training durations.

## Data Availability

The original contributions presented in the study are included in the article/Supplementary Material, further inquiries can be directed to the corresponding author.
